# A Dual-Band Terahertz Absorber with Two Passbands Based on Periodic Patterned Graphene

**DOI:** 10.3390/ma12183016

**Published:** 2019-09-17

**Authors:** Ximeng Zhang, Weiwei Wu, Chenxin Li, Chang Wang, Yuhong Ma, Zhangbiao Yang, Guang Sun, Naichang Yuan

**Affiliations:** College of Electronic Science, National University of Defense Technology, Changsha 410073, China; zhangximeng17@nudt.edu.cn (X.Z.); lichenxin17@163.com (C.L.); wangchang09@icloud.com (C.W.); 15695583160@163.com (Y.M.); yangzhangbiao17@nudt.edu.cn (Z.Y.); sunguang17@nudt.edu.cn (G.S.); yuannaichang@hotmail.com (N.Y.)

**Keywords:** graphene, metamaterial absorber, passband

## Abstract

In this paper, a dual-band terahertz absorber with two passbands is proposed. The absorber is composed of periodic patterned graphene arrays on the top of a SiO${}_{2}$ substrate and a frequency selective surface (FSS) on the bottom of the substrate. The simulated results indicate that there are two absorption bands (absorption greater than 90%) ranging from 0.54 to 0.84 THz and 2.13 to 2.29 THz. It is almost transparent to incident waves (transmission greater than 50%) below 0.19 THz and between 1.3 and 1.67 THz with a center frequency of 1.49 THz. The absorber has a good tolerance to the transverse electric (TE) and transverse magnetic (TM) polarized wave oblique incidence, and the transmission rate of the passbands remains greater than 50% within 70 degrees. Moreover, the absorption rate of the absorber can be tuned by the chemical potential of graphene. The structure with absorption and transmission properties has potential applications in filtering, sensing, detecting and antenna stealth.

## 1. Introduction

Metamaterials (MMs) are artificially designed sub-wavelength electromagnetic (EM) structures. MMs have attracted considerable attention over the past decade due to their exotic properties, such as negative refraction, double negative behavior, left-handed behavior and backward wave propagation [1,2,3,4,5], and their promising applications. The perfect metamaterial absorber (MA) is one of the most widely used groups of MMs [6,7]. Although the perfect MA can perfectly absorb incident EM waves, the bandwidth of the absorption band is narrow and the absorption performance cannot be tuned.

Graphene is a one-atom-thick layer of carbon atoms arranged in a honeycomb lattice. One of the most significant properties of graphene is that its conductivity can be tuned by shifting the electronic Fermi level via electrostatic doping [8,9]. To obtain an adjustable absorption band, graphene-based MAs have been extensively studied. Absorbers with graphene ribbons and disks are proposed in [10,11,12,13,14,15,16,17]. Since these designs have only one resonance of surface plasmon polaritons (SPPs), the absorption bandwidths are narrow. To achieve a wider absorption bandwidth, multi-resonator graphene structures have been proposed in [18,19]. Different absorption peaks can be obtained by resonators with a small difference in one unit cell. Another method is to place different sizes of graphene periodic patterns at different heights in the substrate [20,21,22,23]. The absorbing mechanism is similar to the above design in [18,19]. However, multilayer graphene makes the adjustment of the chemical potential difficult. Graphene absorbers with sinusoidally and elliptically periodic patterns are proposed in [24,25]. Continuous plasmon resonance can be generated on the gradient graphene structures. Therefore, absorbers with wide absorption bands are obtained. For a traditional absorber, EM waves cannot be transmitted because of the ground plane on the underside of the substrate. The inability of EM waves to pass through the absorber limits its application in areas such as filtering and antenna stealth.

In the microwave band, researchers have proposed periodic structures that can absorb and transmit waves in different frequency bands, which are called frequency selective rasorbers (FSRs). FSRs with lower or higher absorption band have been proposed in [26,27,28,29]. However, FSRs can only absorb waves on one side of the passband, and only reflect waves on the other side, which is detrimental to antenna stealth. To overcome this issue, FSRs with an absorption band on each side of the passband have been proposed in [30,31,32]. These FSRs are primarily absorbing EM waves by the lumped resistors loaded on the lossy layer. Since terahertz devices are too small in size compared to microwave devices, the size of the lumped resistor limits its application in the terahertz band. A graphene-based terahertz absorber is proposed in [33]. However, this absorber has only one passband, which is higher than the absorption band.

In this paper, a graphene-based dual-band terahertz absorber with two passbands is proposed and investigated. The structure is composed of a SiO${}_{2}$ substrate, a cross-elliptical graphene periodic pattern in the top of the substrate and a frequency selective surface (FSS) layer in the bottom of the substrate. Unlike the use of lumped resistors in the microwave band, we use cross-elliptical graphene for absorption in the terahertz band. Based on the electric field distribution, we know that the two absorption bands are obtained by electric resonance and magnetic resonance. By adjusting the chemical potential of graphene, we can change the absorption rate of the two absorption bands. Meanwhile, the transmission rate of the two passbands is hardly affected by the chemical potential. The performance of the structure under oblique incidence has also been investigated, and the results show insensitivity to an incident angle within 50 degrees. The structure with absorption and transmission properties has potential applications in filtering, sensing, detecting and antenna stealth.

## 2. Structures and Methods

**Figure 1:**
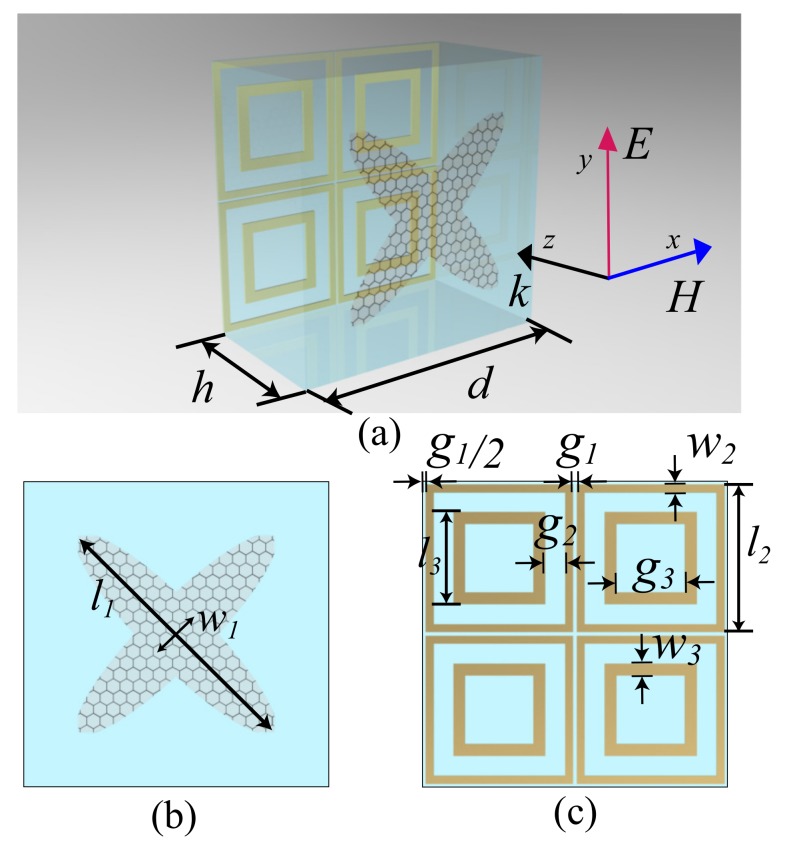
(**a**) Unit cell of the absorber; (**b**) Unit cell of the graphene layer; (**c**) Unit cell of the frequency selective surface (FSS) layer; (structural parameters: *d* = 95, *h* = 54, $l_{1}$ = 43, $w_{1}$ = 9.5, $l_{2}$ = 47, $l_{3}$ = 30, $w_{2}$ = 2.25, $w_{3}$ = 3.75, $g_{1}$ = 0.5, $g_{2}$ = 6.25, $g_{3}$ = 22.5. Unit: μ*m*).

The schematic of the structure is shown in Figure 1. The structure is composed of a SiO${}_{2}$ substrate with a relative permittivity of 3.9 [34], a graphene periodic pattern in the top of the substrate and an FSS made of gold with a conductivity of $4.56\times 10^{7}$ S/m in the bottom of the substrate [35]. The FSS layer is a double-square-loop array [36]. Four FSS units correspond to one graphene unit to obtain the passbands. The graphene layer unit cell is a cross-elliptical graphene pattern. $l_{1}$ represents the major axis of the ellipse, and $w_{1}$ represents the minor axis of the ellipse. The surface conductivity of graphene can be tuned by chemical doping or bias voltage.

The surface conductivity of graphene can be expressed as [37]:

(1) $$\sigma \;=\;\sigma_{inter}+\sigma_{intra}$$

where $\sigma_{inter}$ and $\sigma_{intra}$ are the interband term and intraband term of the graphene surface conductivity, respectively.

The interband and intraband terms of the graphene surface conductivity can be defined as follows:

(2) $$\sigma_{inter}\;=\;-j\frac{e^{2}}{4\pi \hbar }\mathrm{In}\left[\frac{2\left|\mu_{c}\right|-\left(\omega -j2\Gamma \right)\hbar }{2\left|\mu_{c}\right|+\left(\omega -j2\Gamma \right)\hbar }\right]$$

(3) $$\sigma_{intra}\;=\;-j\frac{e^{2}k_{B}T}{\pi \hbar^{2}\left(\omega -j2\Gamma \right)}\left[\frac{\mu_{c}}{k_{B}T}+2\mathrm{In}\left(e^{-\frac{\mu_{c}}{k_{B}T}}+1\right)\right]$$

where *e* is the electron charge, ω is the radian frequency, *ℏ* is the reduced Planck’s constant, Γ=1/2τ is the phenomenological scattering rate, τ is the electron-phonon relaxation time, $\mu_{c}$ is the chemical potential, $k_{B}$ is Boltzmann’s constant, and *T* is the temperature. The surface conductivity is dominated by the intraband contribution in the terahertz band. In this paper, we assume the initial values of the relaxation time, chemical potential, and temperature to be τ = 0.4 ps, $\mu_{c}$ = 0.6 eV and T = 300 K. The absorption of the absorber can be obtained by $A\left(\omega \right)\;=\;1-R\left(\omega \right)-T\left(\omega \right)$, where $A\left(\omega \right)$ is the absorption, $R\left(\omega \right)$ is the reflection and $T\left(\omega \right)$ is the transmission.

## 3. Results and Discussion

**Figure 2:**
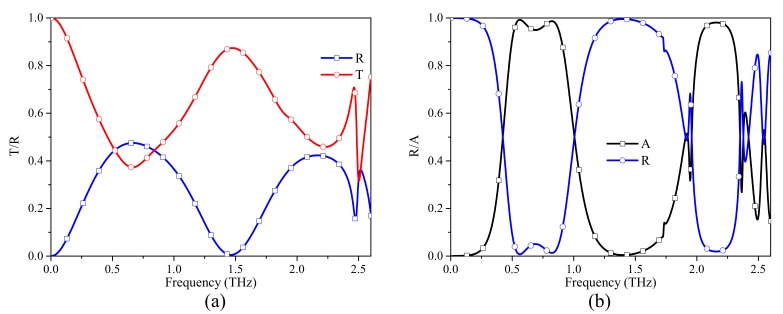
Transmission, reflection and absorption spectra of the structure when $\mu_{c}$ = 0.6 eV and τ = 0.4 ps: (**a**) without the FSS layer; (**b**) replacing the FSS layer with a ground plane.

**Figure 3:**
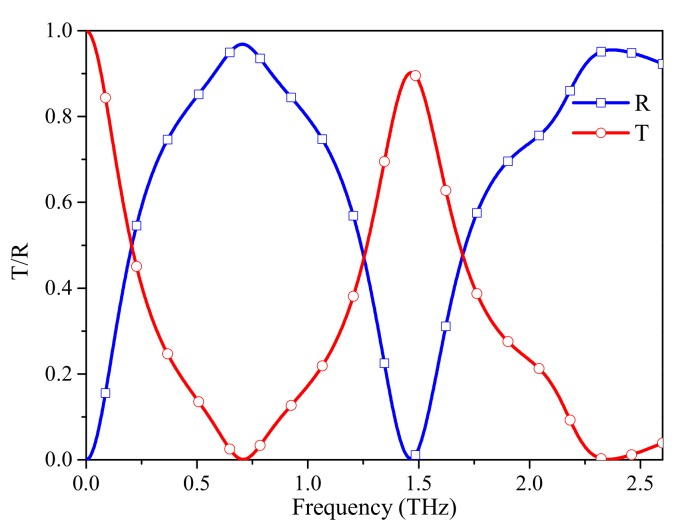
Transmission and reflection spectra of the FSS.

In this study, the numerical simulation results are obtained by the frequency domain finite element method (FEM) solver of the CST Microwave Studio. Periodic boundary conditions are assigned along both the x-direction and y-direction while floquet ports are assigned along the z-direction. Local mesh refinement and adaptive mesh refinement are adopted to enhance the accuracy of the numerical simulation. In the simulation, graphene is modeled as an equivalent 2D surface impedance layer without thickness. Figure 2a shows the transmission and reflection spectra of the structure without FSS. Two transmission windows are obtained: a low-pass band and a bandpass at 1.49 THz. EM waves can pass through the graphene layer with low loss. The insertion loss of the second transmission window is due to the finite surface impedance at this frequency point. Figure 2b shows the absorption spectrum of the structure with a ground plane on the bottom of the substrate. As shown in Figure 2b, there are two absorption bands (absorption > 0.9) at 0.5–0.9 THz and 2.02–2.3 THz. To provide the passbands and reflection bands to the structure, a double-square-loop FSS is introduced. The transmission and reflection spectra of the FSS are shown in Figure 3; the transmission windows of FSS are identical to that of graphene. The two reflection band frequencies of FSS are near the absorption band frequencies of graphene, so the FSS is equivalent to a ground plane at the absorption bands.

**Figure 4:**
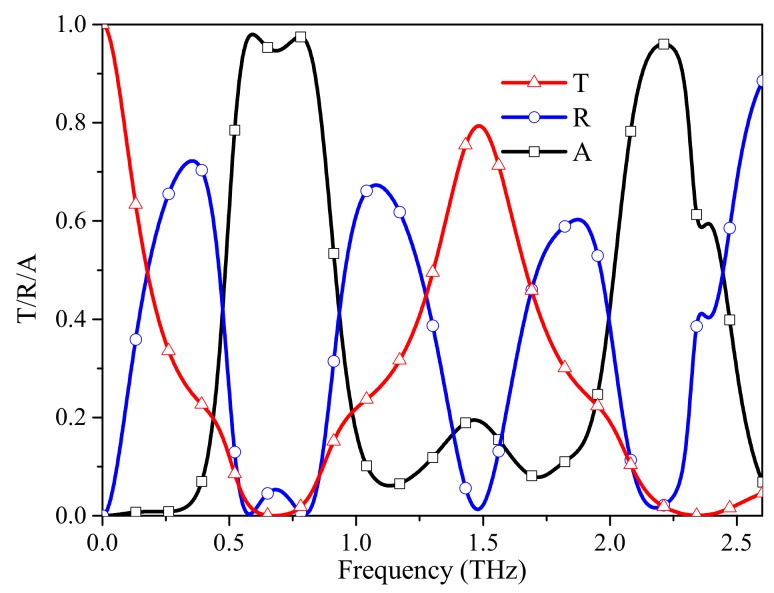
Transmission, reflection and absorption spectra of the absorber when τ = 0.4 ps and $\mu_{c}$ = 0.6 eV.

Figure 4 shows the T/R/A spectra of our absorber when τ = 0.4 ps and $\mu_{c}$ = 0.6 eV, which combines the characteristics of frequency selection and wave absorption. A low-passband is obtained up to 0.19 THz (T > 0.5), and a bandpass band is obtained at 1.49 THz with a bandwidth of 1.3–1.67 THz and a transmission rate of 80%. Compared with that in Figure 2b, the reflection rates near the transmission windows are greatly reduced. There are two absorption bands with 90% absorption rates ranging from 0.54 to 0.84 THz, and from 2.13 to 2.29 THz with normalized bandwidths of 43.5% and 7.2%, respectively. Additionally, both absorption bands have narrower absorption bandwidths than the absorber with a ground plane which is shown in Figure 2b, because the FSS cannot be equivalent to an ideal ground plane (R = 1) in the reflection bands.

**Figure 5:**
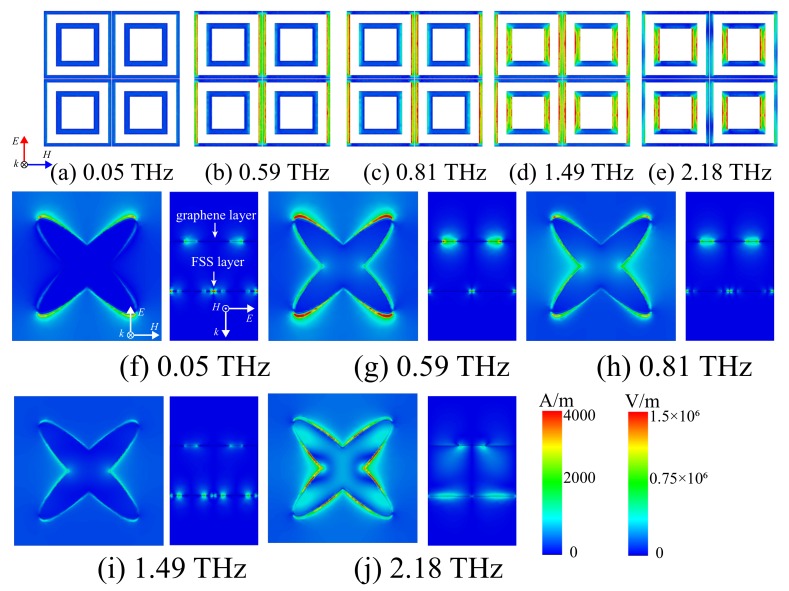
Surface current distribution of the FSS layer at (**a**) 0.05 THz, (**b**) 0.59 THz, (**c**) 0.81 THz, (**d**) 1.49 THz and (**e**) 2.18 THz. Electric field distribution of the absorber at (**f**) 0.05 THz, (**g**) 0.59 THz, (**h**) 0.81 THz, (**i**) 1.49 THz and (**j**) 2.18 THz.

To clarify the physical mechanism of our design, the surface current distribution of the FSS layer and the electric field distribution of our absorber are investigated in Figure 5. In Figure 5d, current flows through the arms of the inner and outer rings parallel to the direction of the electric field, which means that the parallel resonance is generated by the two loops, and a passband is obtained. The current flows in the arms of the outer loop in Figure 5b,c, while in the arms of the inner loop in Figure 5e. This shows that series resonance occurs on both sides of the passband at 1.49 THz. In Figure 5a, the current hardly flows in the FSS layer at 0.05 THz because in the low frequency, the LC series circuit is equivalent to a low-pass filter in low frequencies. Figure 5g,h displays the electric field distribution of the first absorption band. Due to the localized surface plasmon resonance (LSPR) achieved by the gradient width of the elliptical graphene pattern, the electric field of the graphene layer is confined to the edge of the cross-elliptical graphene pattern. The position of the electric field is related to the resonant frequency. When f = 0.59 THz, most of the electric field is confined to the end of the long axis of the ellipses. Electric field is also among the unit cells in the direction parallel to the electric field. When f = 0.81 THz, the electric field mainly exists on the elliptical edge and the gap between adjacent cells in the direction perpendicular to the electric field. Compared with the electric field distribution of the first absorption band, Figure 5j shows that the electric field exists at the edge of the ellipses except the end of the long axis, and it spreads to the interior of the graphene pattern. In addition, the electric field is present in the substrate between the graphene pattern and the gold FSS layer, which demonstrates that the second absorption band is obtained by magnetic resonance between the graphene pattern and the gold FSS layer. As observed from Figure 5i, the electric field in the graphene layer is weak. Thus, the graphene layer has a high impedance, and the EM waves can pass through the graphene layer with high transmission rate. In Figure 5f, the electric field exists between the units parallel to the electric field, because it is equivalent to a low-pass filter in low frequencies.

**Figure 6:**
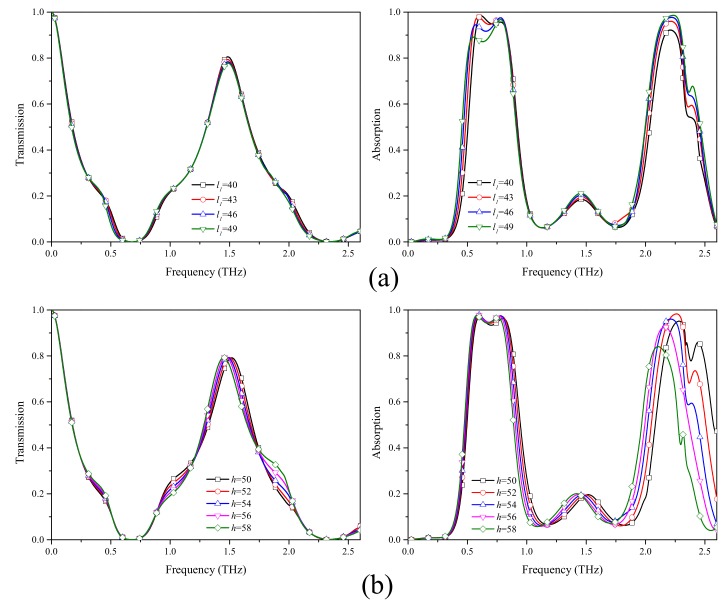
Transmission and absorption spectra of the absorber under conditions of the (**a**) major axis of the ellipse of the graphene pattern $l_{1}$ and (**b**) the thickness of substrate *h*.

To better understand the underlying mechanism, the influence of the length of the major axis of the graphene ellipse and the thickness of the substrate are investigated. As shown in Figure 6a, when $l_{1}$ increases, the transmission rate of the second passband decreases from 0.8 to 0.78. The first peak of the lower absorption band slightly drops and red-shifts. However, the second peak of the lower absorption band remains almost constant. The reason is that the electric field at the first peak is more dependent on the major axis than that at the second peak. As for the higher absorption band, the absorption rate rises with the increase of $l_{1}$, because the electric field spreads from the gap to the interior of the graphene pattern. As shown in Figure 6b, both absorption and transmission spectra show a red shift when the substrate thickness of the substrate increases. It is worth noting that the higher absorption band is more sensitive than the lower band. It rises first and then drops sharply. This is because the thickness of the substrate has great influence on the electric field of the substrate caused by magnetic resonance.

**Figure 7:**
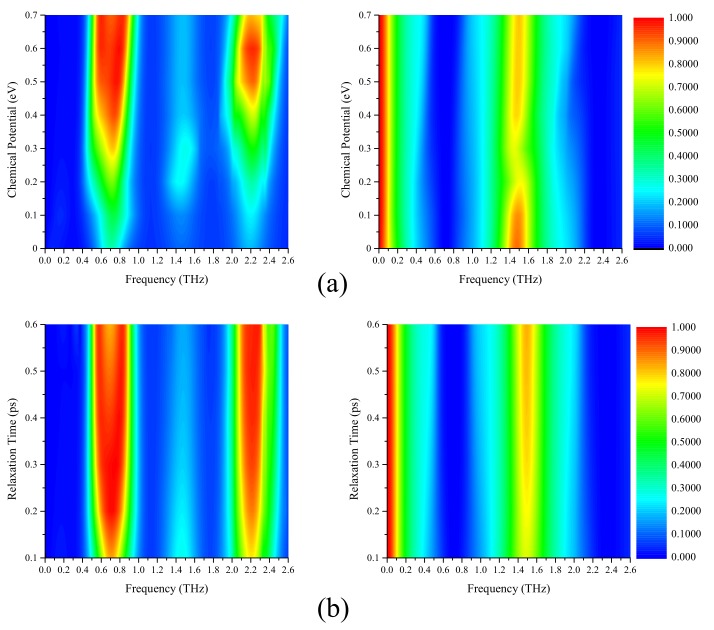
Absorption and transmission spectra for different (**a**) chemical potentials, and (**b**) relaxation times.

The transmission and absorption spectra with different chemical potentials are shown in Figure 7a. When the chemical potential increases, the absorbing bandwidths and absorbing rates of the lower and higher absorption bands increase. When $\mu_{c}$ = 0.6 eV, the charge carriers approach saturation, so the bandwidth of the higher absorption band reaches the maximum at $\mu_{c}$ = 0.6 eV and followed by a decreasing trend. The transmission rate of the second passband initially decreases, subsequently increases, and is maintained at greater than 0.7. Figure 7b shows the variation of the absorption and transmission spectra at different relaxation times. With the increase in relaxation time, the bandwidths of the absorption bands and passband increase. When τ is greater than 0.4 ps, the center absorption rate of the lower absorption band decreases, but the absorption rate of the higher absorption band remains unchanged. It is worth noting that the low-passband hardly changes with chemical potential and relaxation time, because its frequency is too low compared to the operating frequency band of the absorber.

**Figure 8:**
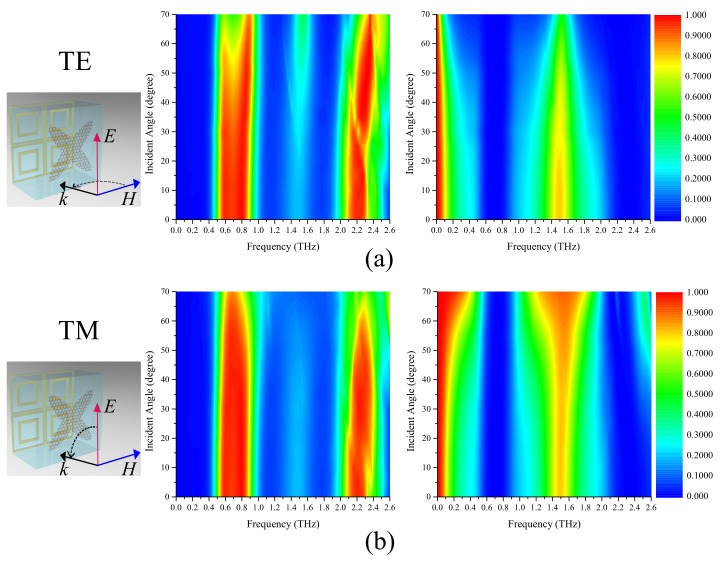
Absorption and transmission spectra for different incident angles under (**a**) transverse electric (TE)-polarized and (**b**) transverse magnetic (TM)-polarized waves.

Furthermore, the performance of our absorber under oblique incidence is investigated in Figure 8. As observed from Figure 8a, the center frequency absorption rate of the lower absorption band slightly decreases with the transverse electric (TE)-polarized incident angle, resulting in the absorbing band being divided into two. This may due to parasitic resonances in the structure at the large incident angle. The higher absorption band shifts to high frequency because the magnetic resonance frequency is affected by the change in the electrical length. The bandwidths of the passbands narrow with the incident angle. However, the transmission rate of the second passband can still maintain 50% under 70 degrees incidence. For the transverse magnetic (TM)-polarized incidence, the lower absorption band maintains a comparable absorption efficiency, and the higher absorption band shifts slightly to a higher frequency. In contrast with the passbands under the TE-polarized incident angle, the bandwidths of the passbands increase with the TM-polarized incident angle. This is because the tangential component of the electric field on the FSS decreases with the TM-polarized incident angle, so the two reflection bands of the FSS become narrower and the passband between the two reflection bands becomes wider. Overall, our absorber can remain high transmission and absorption rates while being independent of the incident angle up to 50 degrees. To demonstrate the performance of the proposed absorber, performance comparisons with previously reported terahertz graphene absorbers are shown in Table 1. The results show that although the proposed absorber has narrow absorbing bandwidths, it has two absorption bands and two passbands and is dual polarized. Since there is only one layer of graphene, it is easy to fabricate.

## 4. Conclusions

In summary, we have demonstrated a dual-band terahertz absorber based on graphene with two passbands. The absorber is composed of a cross-elliptical graphene layer, a double-loop FSS layer and a SiO${}_{2}$ substrate between the two layers. The two absorption bands are achieved by electric resonance and magnetic resonance. The first passband is a low-passband below the lower absorption band, and the second passband is between the two absorption bands. The absorbing peaks can be regulated by chemical potential while the passbands are nearly independent of chemical potential. Furthermore, the performance of our absorber under oblique incidence is investigated and shows angle-independent and polarization-insensitive properties. The absorber has potential applications in filtering, sensing, detecting and antenna stealth.

## Figures and Tables

**Table 1:** Performance comparison with previous terahertz graphene absorbers.

Ref.	Polarization	Number of Passbands	Normalized ${}^{1}$ Bandwidth	Number of Graphene Layers	
[20]	dual	0	14.7% (4.8–5.56 THz)	3
[21]	single	0	84.6% (4.7–11.6 THz)	3
[25]	single	0	41.3% (2.5–3.8 THz)	1
[33]	dual	1	66.7% (0.5–1 THz)	1
This work	dual	2	43.5%/7.2% (0.54–0.84 THz, 2.13–2.29 THz)	1

${}^{1}$ Normalized absorption bandwidth of 90%.
